# Detection of *Borrelia burgdorferi* sensu stricto in *Amblyomma americanum* ticks in the southeastern United States: the case of selective compatibility

**DOI:** 10.1038/emi.2016.45

**Published:** 2016-05-25

**Authors:** Nataliia Rudenko, Maryna Golovchenko, Kerry Clark, James H Oliver, Libor Grubhoffer

**Affiliations:** 1 Biology Centre, Institute of Parasitology AS CR, Department of Molecular Ecology of Vectors and Pathogens, Ceske Budejovice 37005, Czech Republic; 2 Georgia Southern University, James H Oliver, Jr Institute for Coastal Plain Sciences, Statesboro, GA 30460-8056, USA; 3 Department of Public Health, University of North Florida, Jacksonville, FL 32224, USA

**Dear Editor,**

*Amblyomma americanum* (Linnaeus), the lone star tick, is a major human-biting tick in the eastern, southeastern and midwestern USA.^1^ Because of the high population densities and increased expansion ability, its capacity to transmit multiple pathogens, and its aggressive and liberal feeding behavior, *A. americanum* is emerging as one of the most medically and economically significant tick vectors in the United States.^2^ Involvement of *A. americanum* in transmission of *Borrelia burgdorferi* sensu lato (s.l.) spirochetes in the southeastern USA has been a subject of discussion for >30 years, and a limited number of published studies present rather conflicting results.^3, 4, 5, 6, 7, 8^ Although the results of Schulze *et al.*^7^ support the possible role of *A. americanum* as a vector of *B. burgdorferi*, the latest study of Stromdahl *et al.*^8^ did not reveal the presence of *B. burgdorferi* spirochetes in *Amblyomma* ticks. Earlier, *B. burgdorferi* was detected in 5.4% of *A. americanum* adults and in 3.4% of nymphs, collected in 1984 at a major endemic focus of Lyme disease in New Jersey.^9^ Analysis of cultured pools of ticks and fleas collected by the Texas Department of Health in 1988 and 1989 resulted in isolation of *B. burgdorferi* from three of 354 pools of *A. americanum* (0.85%).^5^ Later analysis of *A. americanum* collected in southeastern Missouri detected spirochetes in 1.9% of questing ticks using indirect fluorescent antibody tests with the monoclonal antibody H5332, followed by verification of indirect fluorescent antibody-positive organisms using PCR with two different sets of *B. burgdorferi*-specific primers, and by Southern blotting.^6^ Attempts to define the vector competency of *A. americanum* were conducted in different laboratories, using geographically distant populations of this tick species and laboratory animal models.^3, 4^ The results revealed an interesting finding. Although an *A. americanum* population originated from Texas was completely refractory to infection with *B. burgdorferi* strain JD1 (*ospC* type C), specimens of the same tick species from Alabama showed overall infection rate of 5%.^3^ The following experiments of Sanders and Oliver Jr^4^ involved two different spirochete strains, *B. burgdorferi* SH2-82 (*ospC* type I) and MI-6, later identified as *B. bissettii*. Laboratory transmission of the selected strains was unsuccessful. However, for the first time, the possible compatibility of genetically variable spirochete strains and *A. americanum* populations was discussed,^4^ leading to the conclusion that genetic variability of some strains may allow their maintenance and transmission by certain populations of *A. americanum.* Our recent findings on *B. burgdorferi* infection in a Florida population of *A. americanum* differ from recently published results,^8^ and suggest that, when compatible spirochete strains meet an appropriate tick population, maintenance and transmission may occur.^4^

Five-hundred ninety flat *A. americanum* ticks (203 females (f), 89 males (m) and 298 nymphs (n)) were collected in July 2013 from vegetation in Tennessee (Σ137=39f/21m/77n), South Carolina (Σ60=40f/19m/1n), Alabama (Σ15=10f/5m/0n), Georgia (Σ226=4f/2m/220n) and Florida (Σ152=110f/42m/0n). Ticks were divided by state of collection, females were analyzed individually, males and nymphs in pools of three and six, respectively. A single pool from Georgia included two males and four nymphs. Pooling of the samples resulted in a total of 282 DNA extractions subjected to PCR analyses. A PCR assay that targets a 496-nt fragment of the *flagellin* (*flaB*) gene with primers designed by Clark *et al.*^10^ was used for primary detection of *B. burgdorferi* s.l. in ticks. Multilocus sequence typing (MLST) that involved amplification and sequencing of *clpX*, *pepX, rplB* and *uvrA* genes,^11^ and multilocus sequence analysis that involved *ospC*, *ospA, flagellin*, 16S ribosomal RNA, 16S–23S internal transcribed spacer and 5S–23S intergenic spacer,^12^ were used for spirochete identification. Total DNA of cultured rodent-originated *B. carolinensis* (strain SCW-21) was used as a positive control. Negative controls (reactions without DNA template) were included in all amplifications. DNA purification steps, PCR and post-amplification analyses were set up in separate areas with all precautions as described earlier.^12^ PCR products of the expected sizes^11, 12^ were excised from agarose gels, purified and sequenced in both directions using the same primers as for PCR.

*B. burgdorferi* s.l. DNA was detected in 13 samples by amplification of the *flaB* gene: five samples from Tennessee (4 (f)+1 (n) pool); one from Georgia ((n) pool) and seven from Florida (7 (f)). The prevalence of *B. burgdorferi* s.l. in *A. americanum* females was 5.4% (11 out of 203) with total infection rate of 2.2% (13 out of 590), a prevalence comparable with earlier studies. Here we present the results of analysis of a total of 21 genomic loci from three *B. burgdorferi* s.l. positive samples, Bb316, Bb324 and Bb327 (Table 1), detected in *A. americanum* females #316, #324 and #327 from Florida. The small amount of sample (total DNA purified from one-half of each tick, as another half was used for cultivation) was a significant limitation in this project. We were not able to amplify all 10 selected loci in each of the three positive samples. Nevertheless, results obtained on nine (Bb316), seven (Bb324) and five (Bb327) analyzed loci provided confirmation that spirochete DNA detected in *A. americanum* female ticks was *B. burgdorferi* sensu stricto (s.s.; Table 1). All sequences obtained in this study were deposited in GenBank, and the assigned accession numbers are shown in Table 1. The *Borrelia* spp. MLST database, hosted on PubMLST (pubmlst.org/borrelia/), was used for spirochete identification. In MLST, sequences of housekeeping genes are assigned as alleles for each sample, and combined alleles define the sequence type (ST), providing an unambiguous characterization of the *Borrelia* strain. The *uvrA* sequences of Bb316 and Bb327 match exactly *uvrA* allele 19 that is represented by a limited group (39 isolates) of North American *B. burgdorferi* s.s. strains distributed in New York, Pennsylvania, Connecticut and Canada (Table 1). Bb316 and Bb327 *rplB* sequences match exactly *rplB* allele 1 that is widely distributed in Europe, Canada and the New England, Middle Atlantic, East North Central, West North Central and South Atlantic regions of the USA, as well as California. The partial allelic profile of Bb316 and Bb327 reliably defined the ST of those strains as ST59; this was additionally confirmed by the exact match of *pepX* and *clpX* sequences in both cases. Another strain detected in *A. americanum*, Bb324, has the same *rplB* allele as Bb316 and Bb327, but differs in a *uvrA* allele that matches exactly allele 1 in the database. *UvrA* allele 1 strains are prevalent in the Middle Atlantic and New England states of the USA (Table 1), and in some Canada provinces in close proximity. An exact match of Bb324 *uvrA* and *rplB* loci with MLST alleles defined its ST as either ST1 or ST58. ST58 differs from ST59 (Bb316 and Bb327) only in the *uvrA* locus. All characterized strains detected in *A. americanum* ticks collected in Florida have the same MLST as strains isolated from *Ixodes scapulari*s or humans from highly endemic lyme disease (LD) regions of the USA.

Analysis of Bb316, Bb327 and Bb324 *ospC* genes showed that detected strains had *ospC* types A and M, not previously detected in either ‘bridge' or ‘maintenance' vectors, or in major rodent hosts, of *B. burgdorferi* in the southeastern USA. Until now, all analyzed *B. burgdorferi* strains from the southeastern USA showed the presence of *ospC* alleles B, G, H and L only.^14^ It is possible that in addition to the major enzootic transmission cycle of *B. burgdorferi* s.l. in the southeastern USA that involves *Ixodid* ticks and rodent hosts,^12^ there might be parallel transmission cycles (separate or overlapping), for restricted *B. burgdorferi ospC* type strains, that involve *A. americanum* and hosts other than rodents, for example, white-tailed deer or wild turkey. It is possible that *A. americanum-B. burgdorferi* interaction in the southeastern USA represents a case of differential or selective compatibility (tick population origin?—spirochete *ospC* type?) as was discussed earlier.^4^ Our recent results indirectly support this possibility.^13, 15^ We isolated two *B. burgdorferi* s.s. strains, M6p and M11p, from plasma samples of Georgia residents.^13^ Both suffered from undefined disorders, had symptoms not typical for LD, and recall multiple tick bites in the area of their residence. Unfortunately, the transmitting tick(s) species was not identified. MLST analysis positioned strain M11p between *B. burgdorferi* ST58 and ST59 strains,^15^ such as Bb324 detected in *A. americanum*. Bb324 and M11p showed an exact match in the *rplB* locus, and revealed just two variable sites in the *uvrA* locus (Table 1).

*A. americanum*–*B. burgdorferi* interaction in the southeastern USA is a complex biological issue that involves multiple vector–host–pathogen-related factors. Further research is warranted to determine if the detected strains, or strains of other *ospC* types, associated with systemic LD (A, I and K) or found in disseminated sites (C, D, N, F and E), are viable and transmissible by *A. americanum*. Our further investigations using a laboratory *A. americanum* colony will continue to assess vector competence of *A. americanum* for *B. burgdorferi.*

## Figures and Tables

**Table 1:** Identification of Lyme disease spirochetes detected in Florida population of *Amblyomma americanum* ticks

**MLST**						**MLSA**					
**Locus** **Sample**	***uvrA***	***rplB***	***pepX***	***clpX***	***ST***	***ospC***	***ospA***	***flagellin***	**16S rRNA**	**16S–23S** **ITS**	**5S–23S** **IGS**
Bb316	KU577579	KU577578	KU577577	KU577576		KU577574	KU577573	KU577572	KU577575	KU577571	
PubMLST	Allele 19	Allele 1	Allele 1	Allele 1	59	Type A					
Allele distribution	USA (CT,NY, PA), Canada	USA (CT,NY, MA,MD,IL, MN,MI,WI, CA), Canada, Europe	USA (CT,NY, PA,VT,WI, MA,ME,MN,CA), Canada, Europe	USA (CT,NY, PA,WI, MI, ME,MN,IN, VT,MA,CA) Canada, Europe							N/A
GenBank best match (strains)	M11p^a^, CA382, B31	M6p^a^, M11p^a^	M11p^a^, M6p^a^, CA382, B31	M11p^a^, B31, M6p^a^,CA382		N12^b^, B31, PWag	HB4, B31, A44S	CA382, B31	CA382, B31, JD1	CA382, B31, SGE03-7^b^	
											
Bb324	KU598201	KT598395				KT598393	KU598199	KT598391	KU598197		KU598198
PubMLST	Allele 1	Allele 1			1 (58)	Type M					
Allele distribution	USA (NY,CT, ME, VT, PA, MA, MD), Canada, Europe^c^	as Bb316	N/A	N/A						N/A	
GenBank best match (strains)	M11p^a^, B31, CA382	M11p^a^, CA382, B31				BTW62^b^, 29805	ZS7, Bol26	CA382, B31, ZS7, TWKM1	CA382, B31, N40		M6p^a^, CA382, B31, N40
											
Bb327	KU598202	KT598396				KT598394	KU598200	KT598392			
PubMLST	Allele 19	Allele 1	NA	NA	59	Type A			NA	NA	NA
Allele distribution	as Bb316	as Bb316 as Bb324									
GenBank best match (strains)	M11p^a^, CA382, B31	M11p^a^, CA382, B31				FCR13^b^, CHRW57^b^, BTW11^b^	GT11, ZS7	CA382, B31, JD1			

Abbreviations: multilocus sequence typing, MLST; multilocus sequence analysis, MLSA; sequence type, ST; internal transcribed spacer, ITS; intergenic spacer, IGS; *Borrelia* spp. MLST database (http://pubmlst.org/borrelia/), PubMLST;

Underlined: GenBank accession numbers assigned to sequences obtained in this study;

N/A- not available;

a *B. burgdorferi* s.s. strains isolated from Georgia residents;^13^

b *B. burgdorferi* s.s. strains involved in earlier *ospC* analysis;^14^

c Germany.
